# Valorization of Okara by Enzymatic Production of Anti-Fungal Compounds for Plant Protection

**DOI:** 10.3390/molecules26164858

**Published:** 2021-08-11

**Authors:** Stefano De Benedetti, Valeria Girlando, Matias Pasquali, Alessio Scarafoni

**Affiliations:** Department of Food, Environmental and Nutritional Sciences, University of Milan, 20133 Milan, Italy; stefano.debenedetti@unimi.it (S.D.B.); girlando.valeria@gmail.com (V.G.)

**Keywords:** okara, bioactive peptides, circular economy, biotechnology, proteolysis

## Abstract

Okara is a soybean transformation agri-food by-product, the massive production of which currently poses severe disposal issues. However, its composition is rich in seed storage proteins, which, once extracted, can represent an interesting source of bioactive peptides. Antimicrobial and antifungal proteins and peptides have been described in plant seeds; thus, okara is a valuable source of compounds, exploitable for integrated pest management. The aim of this work is to describe a rapid and economic procedure to isolate proteins from okara, and to produce an enzymatic proteolyzed product, active against fungal plant pathogens. The procedure allowed the isolation and recovery of about 30% of okara total proteins. Several proteolytic enzymes were screened to identify the proper procedure to produce antifungal compounds. Antifungal activity of the protein digested for 24 h with pancreatin against *Fusarium* and *R. solani* mycelial growth and *Pseudomonas* spp was assessed. A dose-response inhibitory activity was established against fungi belonging to the *Fusarium* genus. The exploitation of okara to produce antifungal bioactive peptides has the potential to turn this by-product into a paradigmatic example of circular economy, since a field-derived food waste is transformed into a source of valuable compounds to be used in field crops protection.

## 1. Introduction

Soybean (*Glycine max*) is one of the main crops produced around the world [1]. Soybean for human consumption is often transformed to obtain different kinds of protein isolates, such as soybean curd or soy milk. Okara is a yellowish fibrous material consisting of insoluble residue remaining after these preparations. Its massive production, about 1.2 kg of okara from 1 kg of soybeans for a total yearly production of 1.4 billion tons [2], poses serious issues regarding its disposal. Despite being the leftover of protein isolation processes, okara still represents a rich source of valuable compounds. On average, the dry matter contains 15.2–33.4% of proteins, 3.8–5.3% free carbohydrate, 8.3–10.9% oil and fat, while most of the dry matter is composed of insoluble fiber. The high protein content makes okara a low-cost source of plant proteins and, potentially, of biologically active peptides [3]. In fact, peptides derived from different sources (food, plants, insects) have been shown to exert several bioactivities in animals and plants [4]. One potential application of these peptides is their use as antimicrobial and antifungal molecules.

Fungi and bacteria seriously damage the growth and yield of crops [5] being responsible for the largest number of plant diseases. For example, *Fusarium* rots and *Rhizoctonia* rots can occur in a wide range of crops [6,7]. In particular, fungi of the genus *Fusarium* are considered economically relevant because they can infect cereals, potatoes, vegetables, *Fabaceae*, ornamental, and forest plants. In addition, some *Fusarium* species also produce mycotoxins both in the field and in stored grains, posing serious threats to animal and human health [7]. Fungi of the genus *Rhizoctonia* cause several kind of diseases on *Solanaceae* and other cultivated plants such as rice, cereals, and sugarbeet [8]. Bacteria of the genus *Pseudomonas* are saprophytes and parasites on plant tissue and surfaces. They are responsible for diseases such as rot, necrosis, and galls [9]. *P. syringae pv.* causes, on different tissues of tomato, olive tree, and other plants, local necrotic lesions of various sizes, from tiny flecks to visible lesions [10,11]; *P. solanacearum* is a bacterium distributed worldwide and it causes systemic wilt on different crops like potato, tobacco, tomato, and banana [12].

Conventional crop protection, based on chemical pesticides, is being revaluated for integrated pest management (IPM) that aims to protect human and environment health. Traditional crop protection is based on ready-to-use tools, while IPM is a dynamic system focused on user needs. To make IPM a successful system, alternatives to conventional pesticides are needed to effectively manage crop pests [13]. The features of the ideal pesticide have changed: it must be highly selective for the target species, must have high efficacy at a low application rate, and low environmental persistence that avoids bioconcentration, biomagnification, and development of resistance.

Plants have been sources of natural pesticides for centuries [14]. In particular, the use of plant extracts is considered a good strategy because they have a minimal environmental impact and are potentially nontoxic for consumers [5]. As a matter of fact, plant’s mechanisms of protection against pathogens include a class of proteins and peptides, called defense-related proteins that represent an important strategy to survive infections. Antifungal proteins and peptides have been found in several plant tissues, mostly in seeds [15]. Indeed, during germination, these are vulnerable to pathogen attacks due to the rupture of the seed coat, which allow the invasion of the storage tissue [16]. Antifungal peptides have been described in many seeds, showing inhibitory action against *Botrytis cinerea, Mycosphaerella arachidicola, Fusarium oxysporum*. *Trichoderma reesei,* and *Candida albicans* [17,18]. These antifungal peptides are considered a bioactive molecule group with broad-spectrum activities and multiple mechanisms of action [18]. Antimicrobial peptides have also been found in soybean [19]; hence, there might be natural bioactive molecules with fungicide activity in okara as well.

In this context, the aim of this work is to describe a rapid and economic procedure to isolate proteins from okara and to produce peptides active against fungal plant pathogens by applying an enzymatic approach. The characterization of okara-derived antifungal bioactive peptides has the potential to turn this by-product into a paradigmatic example of circular economy, since a field-derived food waste is transformed into a source of valuable compounds to be used in field crops protection.

## 2. Results

### 2.1. Screening of Protein Extraction Protocols

**Figure 1:**
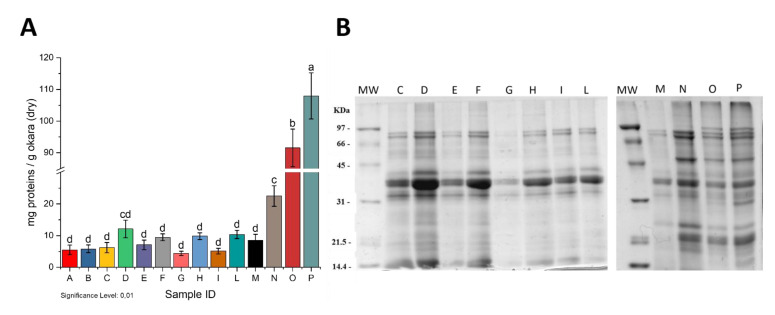
Quantitative (**A**) and qualitative (**B**) analysis of proteins extracted from dry okara in the different tested conditions. Capital letters both in panel A and B refer to Sample IDs described in Table 1. Letters reported above the bars in Panel A (a-d) indicate significant differences (*p* < 0.01) among the samples. SDS-PAGE was run under reducing conditions. The right panel of Figure 1B show isovolumic loadings; left panel show isoproteic loadings. Conditions A and B are not reported in Panel B since they are identical to condition C. MW: Molecular Weight standard marker.

A number of different experimental conditions were tested (Table 1) in order to optimize the solubilization of the okara’s proteins. No quantitative differences of extraction yields (Figure 1A) were observed using non-denaturing conditions, such as water or buffers at pH 6.0, pH 8.0, and pH 10.0, in the presence of high salt concentration (samples A, B, C, and E). The addition of sodium dodecyl sulphate (SDS) as a detergent or a chaotropic agent such as urea at high concentration or reducing conditions allowed to significantly improve the protein solubilization (Figure 1A). No organic solvents were used.

Figure 1B shows the electrophoretic patterns of the different extracts.

Noteworthy, the addition of 5 mM DTT (lanes D, F, H, and L) did not affect the quality of the extracted proteins, although it did enhance the extraction yield (Figure 1A). The addition of 1% SDS allowed not only higher extraction yields (Table 1 and Figure 1A) but also the extraction of more protein species (lanes N, O, and P). The combined use of SDS and reducing agents allowed the extraction of an even higher number of protein species. In this case, the presence of urea did not affect the protein extraction (lanes O and P).

For the subsequent experiments, we choose to extract proteins with 50 mM Tris-HCl pH 8.0 buffer with the addition of 1% of SDS in order to limit the use of chemicals. The extracted proteins were named Okara Total Extract (OTE).

### 2.2. Screening of Proteolytic Enzymes

**Figure 2:**
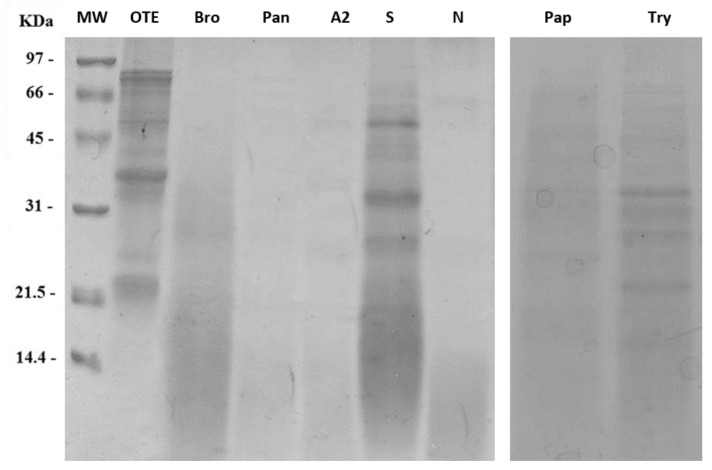
Comparison of digestion with different proteolytic enzymes. OTE stands for Okara Total Extract without any enzyme; Bro: Bromelain; Pan: Pancreatin; A2: Amano A2; S: Amano S; N: Amano N; Pap: Papain; Try: Trypsin. Isovolumic loadings allow to appreciate the different extraction yields obtained during simultaneous digestion and extraction. MW: Molecular Weight standard marker.

Figure 2 shows the electrophoretic separations performed on a 15% SDS-PAGE of the proteolyzed protein extracts obtained with the different tested enzymes. Protein extraction from okara pulp was achieved at different extents depending on the enzyme used, as well as protein digestion. The highest extraction efficiency was obtained with the enzyme Amano S; however, proteolysis was less efficient when compared to other enzymes. Highest degrees of proteolysis were achieved with bromelain and pancreatin. Amano A2 and Amano N proteases caused an extensive level of proteolysis, as almost no protein bands appeared at molecular weights higher than 20 kDa.

The antifungal activity of okara extracts obtained with the aforesaid proteolytic enzymes was initially tested against *F. graminearum* (Supplementary Table S1). Only okara digested with pancreatin showed inhibitory activity, compared to the control. Fungi in the presence of the other extracts grew more than the reference control.

Thus, we decided to focus on pancreatin digestion to optimize protein isolation and digestion protocols.

### 2.3. Preparative Okara Protein Extraction Isolation, Digestion, and Characterization

#### 2.3.1. Protein Isolation and Digestion

Scaling up of the protein extraction procedure performed with 50 mM Tris-HCl pH 8.0 and 1% SDS allowed the recovery of 95 ± 29 mg protein per gram of dry okara, thus comparable to the highest yields obtained in small scale trials (Figure 1A). Total protein content of okara was determined with the Kjeldahl method, resulting 32.9 ± 0.4% of dry matter, thus the extraction protocol applied led to the solubilization of about 30% of total okara proteins. SDS was efficiently removed with the addition of potassium phosphate. Its quantification was performed to evaluate the residual presence of the detergent that could have detrimental effects on the digestion process and the following experimental procedures. SDS residual concentration resulted 0.04 ± 0.01% in Okara Protein Isolate (OPI) samples and 0.004 ± 0.001% in supernatants separated after protein precipitation, confirming that the addition of potassium salts at the end of the extraction procedure led to an almost complete precipitation of the detergent. This allowed to remove in one single centrifugation step the residual biomass as well as the detergent, thus enabling efficient protein solubilization.

**Figure 3:**
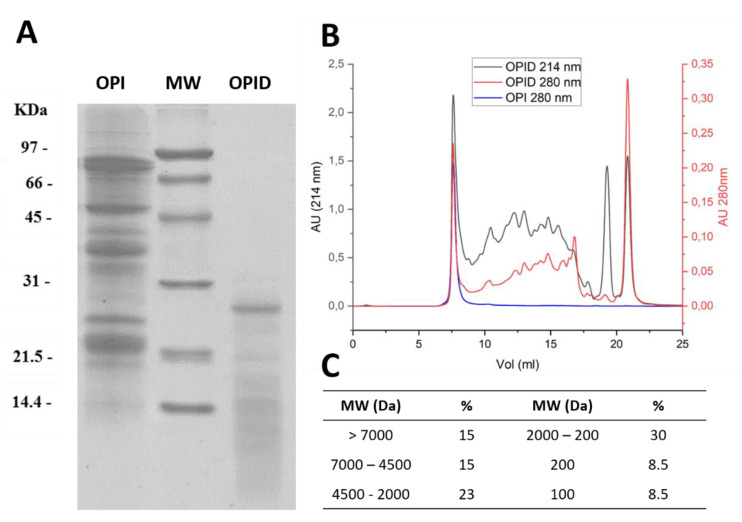
Size distribution of proteins following pancreatin digestion for 24 h. (**A**) 15% SDS-PAGE of Okara Protein Isolate (OPI) and pancreatin digested proteins (OPID). (**B**) Size Exclusion Chromatography (SEC) of OPI (blue) and OPID (red 280 nm; black 214 nm) performed with Superdex 30 increase 10/300 for peptide separation. (**C**) Molecular Weights (MW) distribution calculated according to the calibration with standard MW markers, as detailed in M&M, and expressed as % of the total peaks area.

Precipitation and resuspension of the proteins to obtain OPI allowed the complete recovery of extracted proteins, leaving no detectable proteins in supernatants. The protein content of OPI measured with the Kjeldahl method reached 98.3 ± 0.2%. Overall, extracted, precipitated, and resuspended proteins show molecular weights in the range of 90–20 kDa. Autoclaving of the resuspended OPI to obtain sterilization before digestion did not cause protein precipitation, conversely leading to an increased proteolysis, as is already reported in the literature for soy proteins [20] (data not shown). Digestion performed with pancreatin (1:20 *w*/*w*) over 24 h of incubation led to massive proteolysis, as shown by the increase of protein species with molecular weights below 15 kDa, leaving only few protein species with Mr higher than 20 kDa (Figure 3A).

Gel filtration chromatography allowed the separation of digested peptides according to their size, thus fingerprinting the obtained pancreatin digested proteins (OPID), as reported in Figure 3B. Undigested OPI is shown for comparison. The chromatograms recorded at 214 nm and 280 nm are reported. After extensive proteolysis, a portion of protein species bigger than the column cut off (15% > 7000 Da) was still present (Figure 3A). Digested products distribute with increasing proportions in peptide ranges with decreasing sizes (Figure 3C), calculated as percent peak areas of the total. An equal proportion of species with molecular masses of around 100 and 200 Da potentially representing single amino acids and dipeptides is evident at 214 nm. The pancreatin proteolysis was also analyzed by SEC after 6 h of digestion (Supplementary Figure S1). In this sample, a lower amount of small-sized proteolytic products was observed.

#### 2.3.2. UV Spectra

The collection of spectra in the UV range of OTE allowed the study of its components. The same procedure applied to OTE-derived fractions, OPI and SN, allowing the quantification of their protein contents. Proteins were enriched and separated from other soluble molecules left in the supernatant (SN) at a purity level higher than 98% in OPI through precipitation.

**Figure 4:**
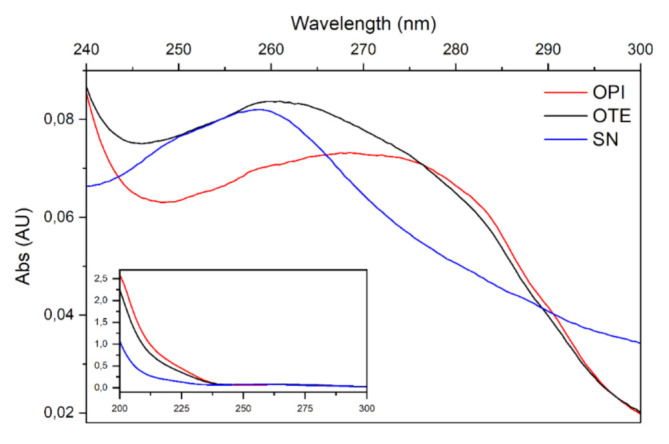
Spectra recorded from 240 to 300 to nm. OTE spectrum (black line) is the result of the sum of two components, one enriched with proteins (OPI, red line), one enriched with molecules with maximum absorption at 260 nm (SN, blue line). In the inset, the full spectrum (200–300 nm) is reported.

Figure 4 makes evident that the spectrum obtained from the OTE sample (black line) is the result of the sum of the spectra relative to different components with different maximum absorption. The OPI spectrum is a typical protein absorption spectrum with a maximum absorption around 280 nm, confirming that the precipitation procedure allowed the enrichment of protein molecules. Conversely, the spectrum of the supernatant sample is devoid of this component, presenting a sharper peak of absorption around 260 nm, a wavelength at which many components present in plant extracts, such as nucleic acids, polysaccharides, xanthines and polyphenols, are absorbed.

#### 2.3.3. Trypsin Inhibitory Activity (TIA)

**Figure 5:**
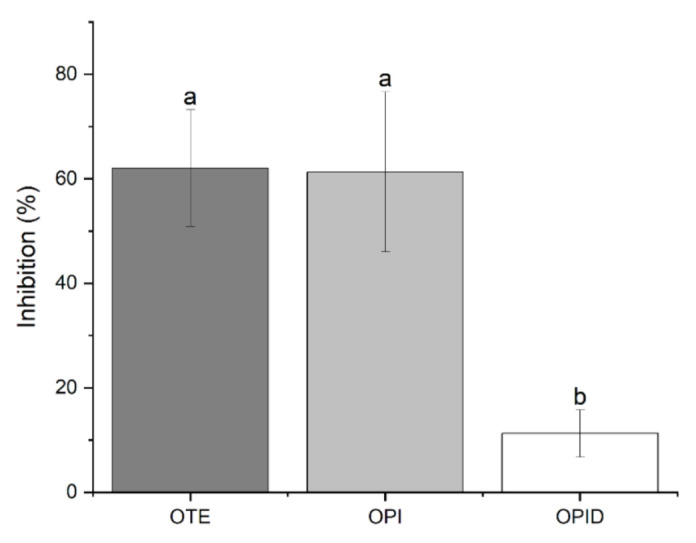
Trypsin inhibitory activity of okara-derived samples. The graph reports the % reduction of trypsin activity in the presence of different tested samples. Significant differences (*p* < 0.01) are indicated by different letters (a–b) at the top of the bars.

Soybean is known to contain protease inhibitors like the Kunitz and the Bowman-Birk trypsin inhibitor [21]. Even if soybean transformation includes thermal treatments, it has been reported that TIA may vary in different extents in the final processed products [22]. Thus, it is likely that in okara residue, anti-tryptic activity could be present, potentially interfering with pancreatin digestion. Results reported in Figure 5 show a comparable TIA for undigested samples. Furthermore, isolation of protein fraction achieved in OPI sample did not alter the extent of the inhibitory activity if compared to OTE, excluding that the reported activity could be related to other non-protein components of the total extract. Despite the presence of protease inhibitors in the undigested sample, pancreatin activity was not affected. Furthermore, the inhibitors themselves reduced their activity from 60% to 10%. Autoclaving samples prior to digestion reduced TIA as well (data not shown), contributing to enhancement in protein digestion performed by pancreatin.

### 2.4. Antimicrobial Activity of Okara Digested Proteins

**Figure 6:**
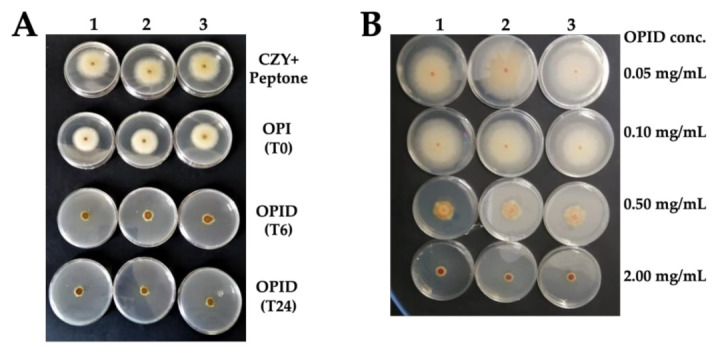
Antimicrobial activity of OPI and OPIDs on *Fusarium graminearum*. (**A**) Samples were tested at a concentration of 0.5 mg/mL. (**B**) The OPID (T24) sample was tested at different concentrations. *F. graminearum* grown in CZY medium added with equivalent peptone concentration was set as reference. OPI (T0) represents undigested okara proteins, while OPID (T6) represents 6 h digested proteins and OPID (T24) represents 24 h digested okara proteins. Three experimental replicas (1, 2, and 3) are reported.

OPID was tested against *F. graminearum* (Figure 6), *F. oxysporum*, *F. verticilloides*), and in *R. solani* (Supplementary Figure S2)

**Figure 7:**
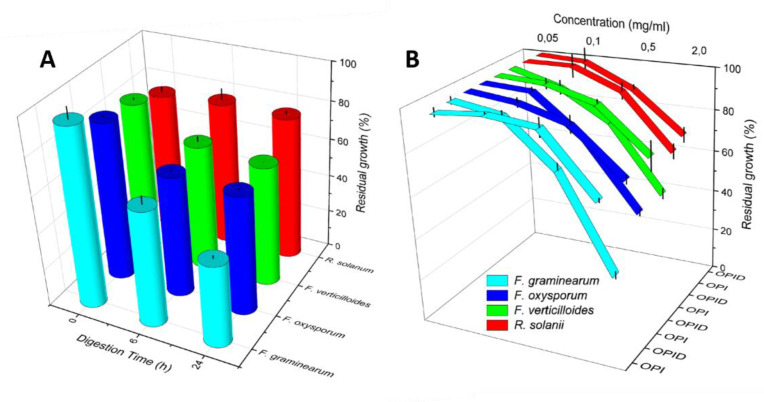
Residual growth of *Fusarium* spp. and *R. solani* following OPI and OPID incorporation in the medium. Panel (**A**) Time dependence of inhibition is tested with OPID samples digested for 6 h and 24 h. Panel (**B**): Dose response of inhibition tested with increasing amounts of 24 h OPID. Error bar represents standard deviation.

OPID obtained after 6 and 24 h showed some inhibitory activity, compared to OPI, as shown in Figure 7A. However, despite an observed increasing trend of inhibition, which suggests that the active molecule(s) were indeed generated through proteolysis, statistically significant differences were evident between samples digested for 6 and 24 h only in *F. graminearum* (Supplementary Table S2). The maximum level of inhibition was reached at 24 h for all the strains of genus *Fusarium * (Figure 6 and Supplementary Figure S2). However, in Rhizoctonia solani, no significant differences in fungal growth were detected between samples treated with either OPI or OPID.

Controls with inactivated enzymes, SDS concentrations, and different pH conditions were tested, showing no effects on *Fusarium * spp. and *R. solani* growth; thus, the effect of fungal growth inhibition was attributed to the proteins digested with the enzyme pancreatin.

To test dose-dependence, OPID was incorporated at different concentrations (0.05–2.00 mg/mL) in *Fusarium * spp and *R. solani* colture media. At a concentration of 0.05 mg/mL, no growth inhibition occurred (Figure 7B), while when OPID concentration was increased, the growth of *Fusarium * spp. was gradually inhibited, indicating a dose response correlation of fungal growth inhibition (Figure 7B, Supplementary Table S3 and Figure S2). No different growth of *R. solani* treated with OPI and OPID was detected.

OPID and supernatant were tested for their antimicrobial activity on Pseudomonas solanacearum and P. syringae, too. OPID exerted an inhibition on the growth of these bacteria of about 80% on both strains (Supplementary Figure S3). The maximum inhibitory activity was detected at 2 mg/mL, while at a lower concentration, the extract did not show any inhibition. In this case as well, supernatant (SN) fraction, leftover of the protein precipitation process, was tested since despite being devoid of proteins, it can still possess interesting antimicrobial compounds. SN indeed showed an inhibitory activity on both strains of Pseudomonas, even if at a lower extent, when compared to the digested protein fraction (44% of growth inhibition).

## 3. Discussion

Reduction of by-products and food wastage to enable full use of natural resources is one of the main challenges the industrialized world faces nowadays. Okara poses, at present, significant disposal issues. Because of the high protein content, it is a by-product interesting and exploitable from a biotechnological point of view. Our small-scale protein extraction trials provided a glimpse into the complexity of the molecular interactions occurring among okara proteins, highlighting a tricky matrix. The process from soybeans to okara likely has detrimental effects on protein stability and solubility [23], hampering the use of this by-product as a source of proteins and peptides. The thermic treatment faced by okara during its processing led to inter-protein crosslinks, mediated by disulphide bridges. This can be deduced since in all conditions tested, the addition of a reducing agent doubled the extraction yield in almost all the cases. The simultaneous use of reducing agents, detergents, and chaotropic substances enhanced the solubilization of okara proteins (Figure 1A). The procedure we finally adopted is a fair compromise between yields and use of large amounts of urea, the presence of which is detrimental for subsequent steps, including the production of peptides by proteolytic enzymes. Our results provide insights into the complex interactions that take place between the different constituents of this raw material, those that can affect the biochemical and enzymatic treatments applied for its transformation. Among the seven proteases we used, pancreatin proved to be the most suitable, as it exhibits high levels of proteolysis that, above all, led to a significant production in antimicrobial activity. On the contrary, the use of the other enzymes resulted in an increase in microbial growth, probably caused by the release of single amino acids that may act as a nutritional supplement.

From both the experiments reported in Figure 6 and Figure 7, it is evident that OPI itself, at different extents, exerts an inhibitory activity on mycelial growth in dependence either of the fungal strain or the protein concentration. It is reported that trypsin inhibitors may exert antifungal activity as a plant defense response [24,25,26]. Thus, the dose-response dependent inhibition activity of OPI samples is likely attributable to TIA. Meanwhile, it appears unlikely that the activity shown by OPID could be related to the same mechanism since a significant decrease of TIA because of the proteolytic digestion process has been demonstrated. Finally, the selective effect of OPID on the *Fusarium* strains, in particular F. graminearum, is noteworthy, while *R. solani* growth is partially inhibited by the undigested sample, but this inhibition does not increase following protein digestion.

We developed a rapid, economic, and scalable method of protein extraction and digestion with pancreatin to produce peptides active against pathogens with a relevant economic interest, such as fungi belonging to the genus *Fusarium* and bacteria like *Pseudomonas*, which can infect many crops.

We believe that the experimentation we have carried out can be a realistic starting point for the implementation of a production process that transforms okara into a low environmental impact crop protection product. Nowadays, several strategies are used for the control of fungal and bacteria growth by chemical treatments [27]. New active substances need to be investigated to find compounds that can effectively fight pathogens and have minimal impact on human beings, animals, and the environment [14].

The production and characterization of okara-derived antifungal bioactive peptides, thus, has the potential to turn this by-product into a paradigmatic example of circular economy, since a field-derived by-product is transformed into a source of valuable compounds to be used in field crops protection.

Finally, these results lay the foundation for further experimentation aimed at testing the peptides produced in vivo using model plants and for final biochemical characterization with the aim of establishing the primary structure of the active peptide(s).

## 4. Materials and Methods

### 4.1. Screening of Protein Extraction Methods

Okara pulp (kindly provided frozen by Valsoia, Serravalle Sesia) was milled with liquid nitrogen until obtaining a uniform granulometry and either stored at −80°C (wet okara) or lyophilized (dry okara).

All chemicals used were purchased from Sigma-Aldrich (St. Louis, MO, USA). Different combinations of solvents (dH_2_O, 50 mM phosphate Buffer pH 6.0, 50 mM Tris-HCl pH 8.0, 50 mM carbonate Buffer pH 10.0) and additives in different concentrations (500 mM NaCl, 5 mM DTT, SDS, Urea) in a total volume of 10 mL (1:25 *w*/*v* ratio with lyophilized okara) were tested to solubilize proteins at room temperature stirring for 3 h. Supernatants were collected after 15 min centrifugation at 12,000 rpm and protein quantification was performed with Bradford assay [28]. Extractions were performed in triplicate.

### 4.2. Screening of Proteolytic Enzymes

Wet okara pulp was suspended in 50 mM Tris-HCl pH 8.0 buffer (1:5 *w*/*v*). The enzymes were then tested with the respective ratios: trypsin (Sigma-Aldrich, St. Louis, MO, USA) 50 µg/g wet okara, papain (Sigma-Aldrich, St. Louis, MO, USA), bromelain (Sigma-Aldrich, St. Louis, MO, USA) 1 U/g wet okara, pancreatin (Sigma-Aldrich, St. Louis, MO, USA), Amano A2, S, and N (Amano Enzyme, Inc., Nagoya, Japan) 1 mg/g wet okara. Incubation was performed for 2 h at 25 °C in all the cases, except for trypsin and papain whose incubation lasted for 4 h. Enzymes were inactivated with the addition of NaOH; the obtained proteolyzed okara was centrifuged at 16,000 ×*g* 90 min at 4 °C, and the supernatant filtered. Okara Total Extract (OTE) was obtained as reference by resuspending wet okara pulp in 50 mM Tris-HCl pH 8.0 buffer (1:5 *w*/*v*), without the addition of any enzyme, incubating it for 2 h at 25 °C. The product was finally heated at 65 °C for 30 min to denature enzymes, which were neutralized with HCl and filter sterilized (Millex-GV Filter, 0.22 µM, Millipore, Burlington, MA, USA).

### 4.3. Okara Protein Extraction Isolation, Digestion, and Haracterization

#### 4.3.1. Okara Protein Isolate Preparation

For large scale protein extraction, milled wet okara pulp was suspended (1:5 *w*/*v*) in 50 mM Tris-HCl pH 8.0 and SDS 1% *w*/*v* stirring at 25 °C for 3 h. To precipitate free KDS 0.1 vol of 1 M Potassium Phosphate Buffer pH 8.0 was added stirring at 25 °C for 30 min as in Carraro et al. [29] with slight modifications. The slurry was then centrifuged 16,000× *g* 90 min at 4 °C, the supernatant was filtered with Whatman^®^ quantitative filter paper Grade 42 (Whatman, Maidstone, UK), and the Okara Total Extract (OTE) was collected. To achieve protein precipitation, 5 M HCl was added until reaching pH 4.5 and an incubation overnight at 4°C was performed. After centrifugation at 16,000× *g* for 30 min, pelleted Okara Protein Isolate (OPI) was separated from its Supernatant (SN), washed twice with distilled water, and lyophilized. For all the further described procedures, OPI was resuspended in 50 mM Tris-HCl pH 8.0.

#### 4.3.2. Okara Protein Isolate Digestion

Once resuspended in 50 mM Tris-HCl pH 8.0, Okara Protein Isolate (OPI) was autoclaved at 121 °C for 15 min to obtain sterilization. Pancreatin from porcine pancreas (Sigma-Aldrich, St. Louis, MO, USA) was dissolved in 1 mM HCl at 5 mg/mL concentration, filter sterilized (Millex-GV Filter, 0.22 µm, Millipore, Burlington, MA, USA), and added to the resuspended proteins with 1:20 (*w*/*w*) proportion. Digestion was performed at 25 °C for 24 h and stopped by heating the solution at 65 °C for 30 min, thus obtaining the Okara Protein Isolate Digested fraction (OPID). Samples after 6 h of digestion were withdrawn and treated with the same procedure in order to control OPI digestion kinetics.

#### 4.3.3. Residual SDS Quantification

Quantification of eventual SDS remaining in Okara Protein Isolate samples was performed with slight modifications of the method described by Žilionis, based on Mukerjee’s photometric method [30]. Briefly, 100 µL of each sample were incubated with 400 µL of cold acetone (Sigma-Aldrich, St. Louis, MO, USA) for 1 h at −20 °C, then centrifuged 10,000× *g* 10 min. 125 µL were then transferred and 1 mL of a solution of 10 µg/mL methylene blue (Carlo Erba, Cornaredo, Italy) in 10 mM HCl, 200 µL of CHCl_3_ were added and the samples were thoroughly vortexed. After 10 min incubation at room temperature, a centrifugation step at 2000 g was performed for 10 min. Samples were left 10 min at room temperature, then 800 µL of the upper aqueous layer were transferred to a plastic cuvette and absorbance read at 655 nm against a blank sample. A standard curve was built with the same procedure with SDS standard solutions at different concentrations between 0.0025% and 0.025%. For each sample, the mean absorbance was subtracted to the mean absorbance of the blank solution to obtain a positive correlation curve. Each sample was measured in triplicate.

#### 4.3.4. Spectrophotometric Characterization of Extracts and Protein Quantification

Total okara protein content was determined by the application of the Kjeldahl method [31] onto three replicates. As for extracted and isolated proteins, common colorimetric quantification methods such as Bradford or Lowry proved to be unreliable due to the presence of SDS, even at negligible concentrations [32]. Spectra of the different fractions obtained (OTE, OPI, OPID, SN) were collected between 200 and 300 nm on a Lambda 2 spectrophotometer (Perkin Elmer, Waltham, MA, USA). Since high absorption at 260 nm was observed, protein quantification of OTE and OPI fractions was performed using the correction described by Kalb et al. [33] with the formula:

[P] (mg/mL) = 0.183 × A_230nm_−0.076 × A_260nm_ (1)

Digested samples (OPID) were quantified according to the far UV absorbance as described by Scopes [34] since the previously mentioned method underestimated the protein/peptide content. Lambert-Beer law at 205 nm was applied and molar extinction coefficient ε_0.1%_ was calculated for each sample using the formula:

ε_0.1%_ 205 nm = 27 + 120 × (A_280nm_/A_205nm_) (2)

#### 4.3.5. SDS–PAGE

SDS–PAGE was carried out according to Laemmli [35] on 12% polyacrylamide gel. For peptides separations, 15% polyacrylamide gels were used. Gels were stained by Coomassie Blue G-250 (BioRad, Milan, Italy.) Low-range SDS-PAGE Standards (Bio-Rad, Hercules, CA, USA) were used for the Mr calculations.

#### 4.3.6. Trypsin Inhibitory Activity (TIA)

Trypsin Inhibitory Activity (TIA) was measured according to the standard method described in ISO 14902:2001 [36]. Briefly, trypsin activity was measured by using the synthetic substrate N-Benzoyl-L-arginine 4-nitroanilide hydrochloride (BAPA, Merck, Life Science, Milan, Italy). Trypsin stock solution 0.27 mg/mL was prepared in 1 mM HCl with 5 mM CaCl_2_ and then diluted 1:20 to perform the assay. BAPA working solution was prepared by diluting 1:100 the stock solution (1.5 mM in DMSO) in 50 mM Tris-HCl pH 8.2, 5 mM CaCl_2_. The assay was performed by mixing 0.1 mL of the protein extract (0.5 mg/mL) with 0.1 mL of BAPA working solution and 0.2 mL of water. After 10 min of incubation at 37 °C, 0.1 mL of trypsin solution was added, and the sample was incubated for 10 min at the same temperature. The reaction was stopped by adding 0.1 mL of 30% acetic acid. The absorbance reading at 410 nm was recorded as a measure of the trypsin activity in the presence of sample inhibitors (As). The reaction was also run in the absence of inhibitors by replacing the sample extract with an equal amount of water to have a reference reading (Ar). Corresponding reagent blanks for the sample readings (Abs) and a reagent blank for the reference readings (Abr) were also prepared by adding the acetic acid solution before the trypsin solution. TIA was calculated as inhibition percent:

Inhibition% = (((Ar–Abr)–(As–Abs)) / (Ar–Abr)) × 100(3)

Each experiment was performed in triplicate.

#### 4.3.7. Chromatographic Separations

The protein content of OPI and OPID was analyzed by size-exclusion chromatography with a Superdex Peptide Increase 10/300 (GE Healthcare, Chicago, IL, USA) equilibrated in 50 mM Tris-HCl pH 8.0 onto a Waters Delta 600 HPLC system (Waters Corporation, Milford, MA, USA) equipped with a Waters 2487 dual absorbance reader (Waters Corporation, Milford, MA, USA) set at 214 and 280 nm. 100 µL of samples at the same concentrations were loaded and eluted in isocratic mode with a flux of 0.5 mL/min. Calibration was performed with known Mr standards.

### 4.4. Plant Pathogen Bioassays

#### 4.4.1. Fungal and Bacteria Isolates

To test the inhibitory activity of the proteolyzed samples obtained from okara, three different *Fusarium* species were used (Table 2): *F. graminearum* (Fg 3005) collected in Australia in 2001 from barley, *F. oxysporum f. sp. Lycopersici* (Fgsc 10446) collected in Italy from tomato, and *F. verticilloides* (MUCL43478) collected in California from corn. One strain of *Rhizoctonia solani* was also included. As for bacteria tests, two different species of *Pseudomonas* were used: *P. solanacearum* (Smith) Biotype IV (IPV-2574) and *P. syringeae pv. Tomato* (Okabe) (Young et al.).

#### 4.4.2. In Vitro Tests of Fungal Mycelial Growth Inhibition

In vitro tests of fungal mycelial growth inhibition were performed with the same procedure described below to test different conditions, with the proper described controls. Firstly, assays were performed to screen the effect of the different proteolytic enzymes against *F. graminearum*. Then, digestion kinetics was assayed by testing Okara Protein Isolate (T0) and Okara Protein Isolated digested with pancreatin for 6 h (T6) and 24 h (T24) at a constant concentration of 0.5 mg/mL. Finally, dose-dependent inhibitory activity was assayed by changing the amounts of OPI and OPID digested 24 h (0.05–2 mg/mL) to determine the maximum inhibitory concentration and the concentrations that do not inhibit fungal growth. In these last two cases, inhibitory activity was tested also against *F. oxysporum, F. verticilloides* and *R. solani*.

The biological activity of the treatments was determined by incorporating aliquots of the different okara samples into Czapek medium to obtain the desired final protein concentrations. Czapek medium was prepared as follows: 35 g Czapek nutrient broth (Sigma-Aldrich, St. Louis, MO, USA), 2 g yeast extract (Sigma–Aldrich, St. Louis, MO, USA), and 15 g agar (Sigma–Aldrich, St. Louis, MO, USA) dissolved in 750 mL of water. The media were prepared at 1.3× concentration in order to have the same nutrient composition into the final solution, after addition of the proper volumes of okara samples. Okara samples were sterilized and heated at 50 °C to bring them to the same temperature as the medium and then incorporated in it in the proper proportion. pH was corrected after the addition of okara sample to have the same pH of the controls at a final value of 8.0.

Mycelial disks of different fungi were transferred from the edges of a seven-day-old culture to plates filled with the media containing okara samples. Different control experiments were considered: Czapek medium plus undigested OPI (T0), Czapek medium plus equivalent peptone concentration, Czapek medium plus each single enzyme solution at the same concentration used in the digestion procedures, Czapek medium plus SDS 0.04%. Radial mycelial growth was determined after 36 h by calculation of the average of two perpendicular colony diameters for each replicate. Each experiment was performed in triplicate.

#### 4.4.3. Microplate Assay

Bacteria were grown for 48 h in KB liquid medium (Duchefa Biochemie, Haarlem, NL) at 25 °C to reach a concentration of 10^9^ ufc/mL. A flat bottom 96 microplate was prepared with 4 replicates for each okara sample tested. 150 μL of 1.3 × KB medium, either 40 μL of each okara sample or 40 μL of sterile deionized water for the control, and 10 μL of bacteria suspension (10^7^ ufc) were added in each well. Different concentrations (2 mg/mL; 0.5 mg/mL; 0.1 mg/mL; 0.05 mg/mL; 0.005 mg/mL) of OPI and OPID were then assayed to test the dose-dependent inhibitory activity and to determine the maximum inhibitory concentration and the concentrations that do not inhibit growth. Equivalent peptone concentration solutions were set as reference controls. Supernatant (SN) obtained after protein precipitation was also tested by adding 40 μL of the neutralized solution in each well using a solution of 0.004% SDS as control. Controls to determine medium sterility were added for each condition as well. The microplate was closed and incubated at 25 °C. Absorbance readings were performed at 600 nm three times per day over 2 days. All experiments were confirmed twice.

### 4.5. Statistical Analysis

Statistically significant differences among the different extraction methods tested, in terms of protein extraction yields, and TIA assays were calculated using Tukey’s test for mean comparisons (significance was set at *p*-value < 0.01). Fungal mycelial growth inhibition expressed as % residual growth of colonies in the presence of OPID, with respect to each proper reference sample, were compared by Student’s t-test (significance was set at *p*-value < 0.05). Statistical analyses were performed with the software Origin (Pro), Version 2021b. (OriginLab Corporation, Northampton, MA, USA). The same software was used to generate graphs.

## Figures and Tables

**Table 1:** Extraction conditions (A to P) of proteins from lyophilized okara.

Sample	Solvent				Additives			
ID	ddH_2_O	pH 6.0	pH 8.0	pH 10.0	NaCl 500 mM	5 mM DTT	SDS	Urea
A		x			x			
B				x	x			
C			x		x			
D			x		x	x		
E	x							
F	x					x		
G			x					
H			x			x		
I	x							2 M
L	x					x		2 M
M			x				0.2%	
N			x				1.0%	
O			x			x	1.0%	
P	x					x	1.0%	8 M

**Table 2:** Fungal and bacterial strains used in this work. Fg3005 was kindly provided by D. Gardiner. FGSC= Fungal Genetics Stock Center; MUCL = Mycothèque de l’Université catholique de Louvain; IPV = Istituto Patologia Vegetale di Milano.

Strain Number Collection	Taxonomy
Fg 3005	*Fusarium graminearum*
FGSC 10446	*Fusarium oxysporum f.* sp. *Lycopersici*
MUCL43478	*Fusarium verticillioides*
-	*Rhizoctonia solani*
IPV-2574	*Pseudomonas solanacearum (Smith) Biotype IV*
IPV-2591	*Pseudomonas syringae pv. Tomato (Okabe)* Young et al.

## Data Availability

The data presented in this study are available on request from the corresponding authors.

## Supplementary Materials

The following are available online. Table S1. Screening of inhibitory activity of the *F. graminearum* strain of the proteolyzates obtained with different proteolytic enzymes. Table S2. Time-dependent fungi growth inhibition statistical significance. Table S3. Concentration-dependent fungi growth inhibition statistical significance. Figure S1. Size Exclusion Chromatographies (SEC) of OPID (Okara Protein Isolate Digested). Figure S2. Antimicrobial activity pictures. Figure S3. Antimicrobial activity on *Pseudomonas solanaceum* and *P. syringae*.

## Sample Availability

Samples of the compounds are available from the authors.
