# Supplementary material for: Valorization of Okara by Enzymatic Production of Anti-Fungal Compounds for Plant Protection
Source: Molecules. 2021 Aug 11;26(16):4858. doi: 10.3390/molecules26164858 (PMC8400248; doi:10.3390/molecules26164858)
Supplement: Supplementary file 1 [file molecules-26-04858-s001.zip › molecules-1323277-supplementary.pdf]

Supporting Information

Valorization of okara by enzymatic production of anti-fungal compounds for plant protection.

Stefano De Benedetti [1 §], Valeria Girlando [1 §], Matias Pasquali [1, *] and Alessio Scarafoni [1, *]

Table S1. Screening of inhibitory activity of *F. graminearum* strain of the proteolyzed products obtained with different proteolytic enzymes. Data are reported as percent growth in comparison with untreated control; SD = Standard Deviation.

Fusarium graminearum		
Enzyme	% growth	SD
Pancreatin	90.68	3.27
Trypsin	124.84	3.36
Papain	128.57	11.45
Bromelain	137.89	2.46
Amano N	116.46	4.66
Amano A	130.12	1.94
Amano S	101.86	2.34

Table S2. Fungi growth inhibition statistical significance as mean comparisons of OPI *vs* OPID digested either for 6 hours or 24 hours with pancreatin, established with t-test onto three replicates for each experiment. In bold are reported those values with statistical significance at p < 0.05.

Treatment comparisons	*p-value*			
	F. graminearum	*F. verticilloides*	*F. oxysporum*	*R. solani*
T0 vs T6	**0.0003**	**0.003**	**0.003**	0.141
T0 vs T24	**0.0004**	**0.005**	**0.004**	0.238
T6 vs T24	**0.03**	0.121	0.144	0.566

Table S3. Fungi growth inhibition statistical significance as mean comparisons of OPID *vs* OPI at different concentrations, established with t-test onto three replicates for each experiment. In bold are reported those values with statistical significance at p < 0.05.

Treatment concentration (mg/mL)	*p-value (OPI vs OPID)*			
	F. graminearum	*F. verticilloides*	*F. oxysporum*	*R. solani*
2.00	**0.0001**	**0.007**	**0.0005**	0.21
0.50	**0.01**	0.10	**0.05**	0.09
0.10	**0.05**	**0.03**	0.09	0.21
0.05	0.09	0.33	0.15	0.37

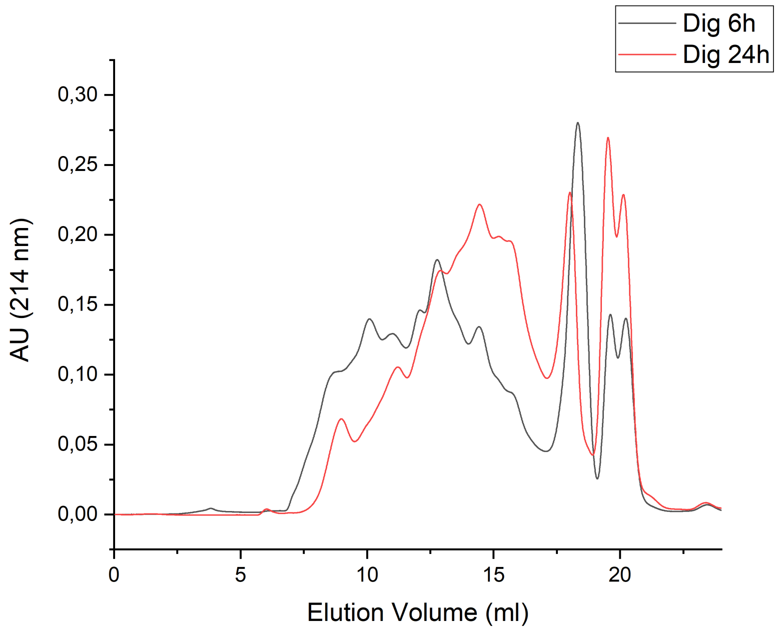

Figure S1. Size Exclusion Chromatographies (SEC) of OPID (Okara Protein Isolate Digested with pancreatin) after 6 hours of digestion (black) and 24 hours (red) at 214 nm, performed with Superdex 30 10/300 for peptide separation. In the 24 hours digestested sample an increase of low molecular weights proteolytic products can be observed.

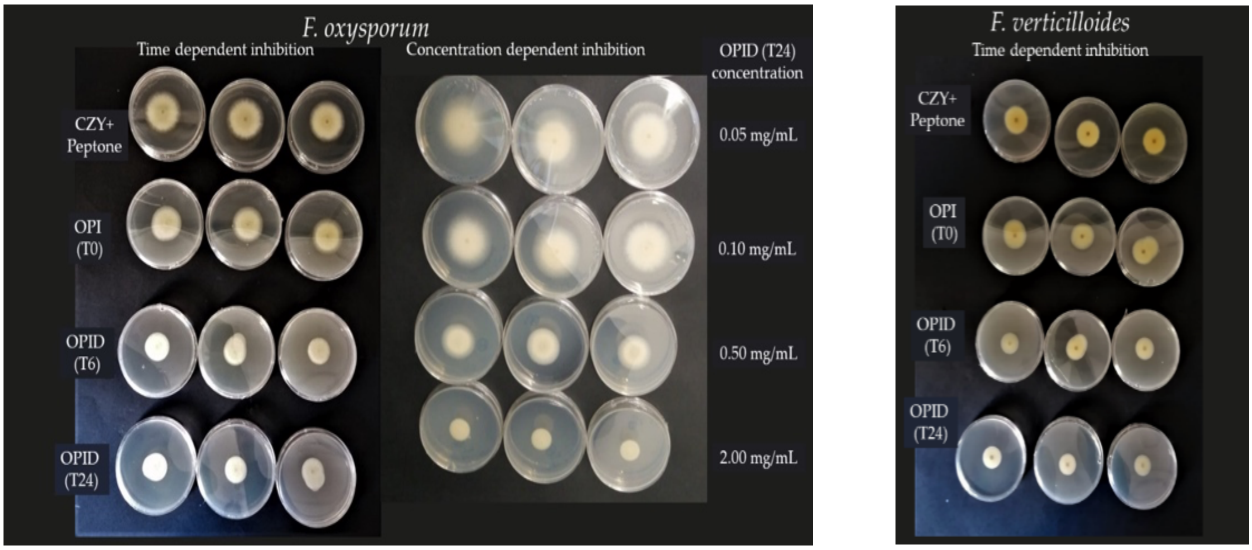

Figure S2. Antimicrobial activity pictures. *Fusarium spp.* time dependent (sample concentration: 0.5 mg/mL) and concentration dependent inhibition (sample: OPID T24). Fungi grown in CZY medium additioned with equivalent peptone concentration were set as reference. OPI (T0) represents undigested okara proteins, while OPID (T6) represents 6 hours digested proteins and OPID (T24) represents 24 hours digested proteins.

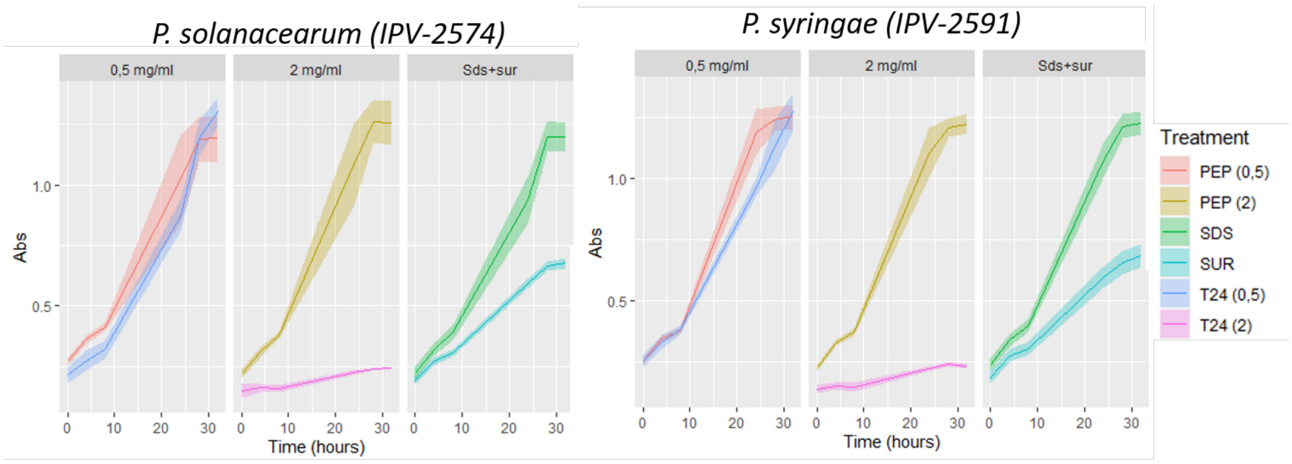

RESIDUAL GROWTH %	*P. solanacearum* (IPV-2574)	*P. syringae* (IPV-2591)
OPID 2 mg/ml	19.35 ± 0.61	19.18 ± 0.96
OPID 0.5 mg/ml	109.28 ± 6.07	101.8 ± 7.32
SN	56.40 ± 2.20	55.72 ± 4.58

Figure S3. Antimicrobial activity on *Pseudomonas solanaceum* and *P. syringae*. OPID at different concentrations and supernatant were tested. Results are reported as residual growth % ± Standard Deviation with reference to peptone control for OPID end to SDS 0.004 % for supernatant. PEP: Peptone control. SDS: Sodium dodecyl sulfate control. SUR: Supernatant. T24: Okara Protein Isolate 24 h Digestion (OPID).
